# Reticulated Platelets and Their Relationship with Endothelial Progenitor Cells during the Acute Phase of ST-Elevation Myocardial Infarction

**DOI:** 10.3390/jcm11216597

**Published:** 2022-11-07

**Authors:** Nili Schamroth Pravda, Mark Kheifets, Maya Wiessman, Dorit Leshem-Lev, Hana Vaknin Assa, Ran Kornowski, Yeela Talmor-Barkan, Leor Perl

**Affiliations:** 1 Department of Cardiology, Rabin Medical Center, Petach Tikva 4941492, Israel; 2 Sackler Faculty of Medicine, Tel Aviv University, Tel Aviv 6997801, Israel; 3 Felsenstein Medical Research Center, Rabin Medical Center, Petach Tikvah 4941492, Israel

**Keywords:** endothelial progenitor cells, reticulated platelets, thrombosis, STEMI

## Abstract

Introduction: Endothelial progenitor cells (EPC) and reticulated platelets (RP) have central roles in the thrombotic and angiogenetic interactions during ST-elevation myocardial infarction (STEMI). The EPC and RP response in patients with STEMI treated by primary percutaneous intervention (PPCI) has not yet been investigated. Methods: We assessed EPC quantification by the expression of CD133^+^ and CD34^+^, and EPC function by the capacity of the cells to form colony-forming units (CFU) and MTT (3-(4,5-dimethylthiazol-2-yl)-2,5-diphenyl tetrazolium bromide) during the acute phase of STEMI. These measurements were correlated with RP at baseline and after 24 h following PPCI. Results: Our cohort included 89 consecutive STEMI-diagnosed patients enrolled between December 2018 and July 2021. At baseline, there was a strong positive correlation between reticulated platelet quantity and MTT levels (R = 0.766 and R^2^ = 0.586, *p* < 0.001), CD34^+^ levels (R = 0.602, and R^2^ = 0.362, *p* < 0.001); CD133^+^ levels (R = 0.666 and R^2^ = 0.443, *p* < 0.001) and CFU levels (R = 0.437, R^2^ = 0.191, *p* < 0.001). The multiple linear regression showed that levels of MTT (adjusted R^2^ = 0.793; *p* < 0.001), CD34^+^ and CD133^+^ (adjusted R^2^ = 0.654; *p* < 0.001 and adjusted R^2^ = 0.627; *p* < 0.001, respectively) had strong independent correlations with RP response. At 24 h after PPCI, the correlation between RP quantity and EPC markers was not significant, except for MTT levels (R = 0.465, R^2^ = 0.216, *p* < 0.001). Conclusions: In patients with STEMI, higher levels of RP at baseline are significantly correlated with a more potent EPC response. The translational significance of these findings needs further investigation.

## 1. Introduction

Platelets have an integral role in the thrombotic cascade following plaque rupture and the ensuing acute coronary syndrome. (1) Reticulated platelets (RP), as compared to mature platelets, are larger, immature platelets mobilized from the bone marrow. As the youngest subpopulation of platelets, RP are an index of platelet turnover. These RP have amplified thrombotic activity [1].

Endothelial progenitor cells (EPC) are also bone marrow-derived cells that play a vital role in the process of vascular repair and homeostasis. These cells are mobilized to sites of vascular injury and can proliferate and differentiate into endothelial cells. These cells promote re-endothelization and neovascularization, thus promoting endothelial integrity [2]. The depletion or impaired function of EPC is associated with endothelial dysfunction and cardiovascular disease. There are strong interactions between EPC and platelets. Exposure to platelets has been shown to augment the functional properties of EPC in vitro, mediate their recruitment to the location of vascular injury and stimulate EPC differentiation into endothelial-phenotype cells [3,4].

Tissue ischemia, and the ensuing thrombotic milieu of multiple chemoattractants, is a strong trigger for both RP and EPC mobilization [5,6]. Acute myocardial infarction is a unique clinical entity, associated with a profound increase in both EPC and RP response. The magnitude of the EPC response after acute myocardial infarction has been shown to correlate with the extent of cardiac necrosis and has prognostic importance [7]. Various factors, such as glycemic control, reactive oxygen species and inflammatory markers, have been shown to affect the EPC number and functioning in this setting [8] (D’Onofrio et al., 2019). However, the interaction between EPC and RP has not been investigated in patients with STEMI (ST-elevation myocardial infarction). We therefore set out to examine the correlation between the EPC and RP in vivo in the acute phase of STEMI among patients treated using primary percutaneous coronary intervention (PPCI).

## 2. Methods

**Figure 1:**
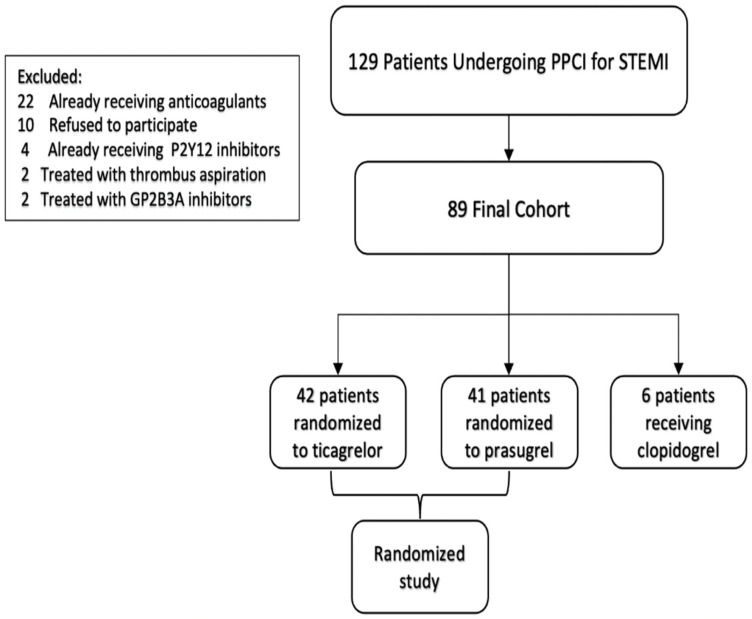
Study flow diagram.

Consecutive patients diagnosed with STEMI undergoing primary percutaneous intervention at the Rabin Medical Center between December 2018 and July 2021 were included in this study. Patients were diagnosed with STEMI when presenting with chest pain and ECG findings of STEMI [9]. These patients were treated with either ticagrelor, prasugrel or clopidogrel. Of these, 83 were randomized to ticagrelor or prasugrel, as part of a clinical trial assessing the impact of each medication on EPC, thrombin generation and RP response (Figure 1). The researchers performing the tests were blinded to the medication group.

Patients were excluded from the trial if they: (a) were already treated with a P2Y12 inhibitor; (b) were treated with anticoagulation for any reason; (c) had recent major gastrointestinal bleeding; (d) were diagnosed with end-stage malignant disease; (e) had any other contra-indication for prasugrel or ticagrelor treatment; (f) had GPIIbIIIa use during the PCI; or (g) were treated with thrombus aspiration during PCI.

A previous diagnosis of coronary artery disease, previous percutaneous intervention (PCI) and coronary artery bypass surgery were not exclusion criteria.

The management of the patients was carried out according to guideline-directed management, with coronary angiography and intervention performed as soon as possible [9]. All patients were treated with a loading dose of prasugrel 60 mg, ticagrelor 180 mg or clopidogrel 600 mg immediately after PCI. The choice of stent, as well as other therapeutic modalities such as mechanical support devices, drug eluting balloons or distal protection devices, were left to the discretion of the primary operator. All stents were implanted with moderate-to-high deployment pressure (12 to 16 atm). All patients received dual antiplatelet therapy with aspirin 100 mg daily and a thienopyridine (clopidogrel, prasugrel or ticagrelor) for at least 12 months after PCI.

All clinical investigations were conducted according to the principles of the Declaration of Helsinki and were approved by the institutional ethics board, and patient consent for participation in this trial was obtained.

### 2.1. Blood Sampling

Whole blood samples were drawn at baseline, prior to the administration of P2Y12 inhibitors or anti-coagulant therapy, through a venous puncture. These samples were then separated to citrated tubes for platelet function assessment, to heparinized tubes for EPC extraction and to EDTA tubes for fluorescence-activated cell sorting (FACS).

EPC, as well as pertinent markers of platelet activation, were assessed at baseline and 24 h after the primary percutaneous intervention. EPC levels were assessed by flow cytometry for the expression of CD133^+^ and CD34^+^. These were assessed as markers of quantification of EPC. The functional aspects of EPC were evaluated by the capacity of the cells to form colony-forming units (CFUs) and the MTT (3-(4,5-dimethylthiazol-2-yl)-2,5-diphenyl tetrazolium bromide) assay, performed to evaluate the viability of the cultured EPC. CD133^+^, CD34^+^ and MTT are presented as the percentage of cells co-expressing these markers. An EPC colony was defined as a cluster of at least 100 flat cells surrounding a cluster of rounded cells. The results were expressed as the mean number of CFUs per field. Reticulated platelets were reported as a percentage of the total platelet levels. Platelet reactivity was assessed with the Accumetrics VerifyNow^TM^ PRUTest^TM^ (24). Platelet function was evaluated at bedside in the catheterization laboratory, while EPC were studied in the laboratory of cardiovascular biology at the Felsenstein Research Center, located at the Rabin Medical Center.

### 2.2. Statistical Analysis

The study size calculation assumed differences of 700 in the AUC of thrombin generation between the two different medications (ticagrelor and prasugrel), and at 80% power, a sample size of approximately 35 patients per group was required to reject the null hypothesis at an alpha level of 0.05. Patients’ characteristics were presented as *n* (%) for categorical variables and as the mean ± standard deviation (SD) or median (interquartile range—IQR) for symmetrically or asymmetrically distributed continuous variables, respectively. Continuous variables following a normal distribution were compared using Student’s *t*-test, whereas those not following a normal distribution are presented as the median and interquartile range and were compared using the Mann–Whitney U test. Categorical variables are reported as counts and percentages. The valid percent was reported. The Pearson correlation was used to explore the continuous relationship between measurements of RP and EPC. EPC were also compared across RP tertiles using analysis of variance testing. Finally, a multiple linear regression was performed to assess the relationship between the RP and the different EPC variables. Included were the following parameters: age, sex, previous coronary artery disease, diabetes mellitus, peripheral arterial disease, prior statin treatment, angiotensin-converting enzyme inhibitors treatment, platelet levels at admission, hemoglobin, creatinine and current smoking.

All tests were conducted at a two-sided alpha level of 0.05 which was considered statistically significant. All statistical analyses were performed using IBM SPSS Statistics for Windows, Version 28.0 (Armonk, NY, USA: IBM Corp., 2021).

## 3. Results

Our cohort included 89 study-eligible STEMI patients enrolled between December 2018 and July 2021. The mean age was 61.9 ± 11.0 years, with the majority (66.3%) being male patients. Baseline characteristics are shown in Table 1.

The laboratory findings are shown in Table 2. The platelet reactivity (as assessed by PRU) and quantity of RP decreased following primary percutaneous intervention (PPCI) and anti-platelet drug initiation, whereas the makers of EPC quantification (CD34^+^, CD133^+^) and function (CFU and MTT levels) increased following PPCI and anti-platelet drug initiation.

**Figure 2:**
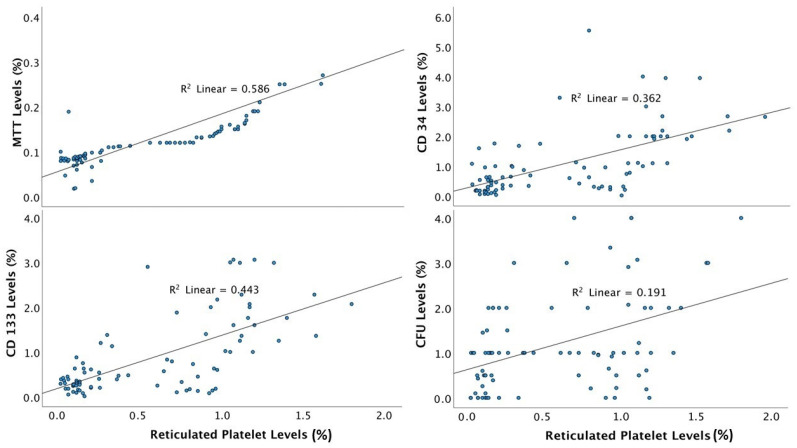
Regression analysis of EPC markers by RP tertiles at baseline assessment. CFU = colony-forming units; MTT = 3-(4,5-dimethylthiazol-2-yl)-2,5-diphenyl tetrazolium bromide.

Table 3 and Figure 2 show a regression analysis of EPC markers by RP tertiles at baseline assessment. The EPC markers, both of quantification and function, were all significantly more pronounced in the highest RP tertiles compared with the middle and lower tertiles.

**Figure 3:**
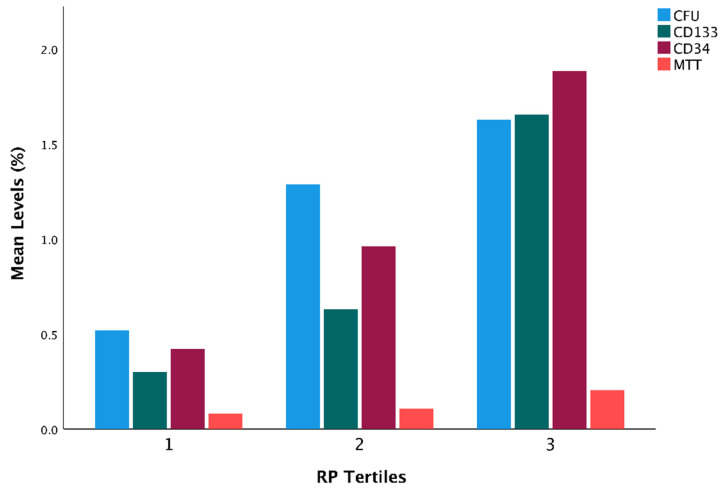
Correlation for the four EPC markers and RP quantity at baseline. CFU = colony-forming units; MTT = 3-(4,5-dimethylthiazol-2-yl)-2,5-diphenyl tetrazolium bromide; RP = reticulated platelets.

Figure 3 and Table 4 show a strong positive correlation for the four EPC markers and RP quantity. In the continuous analysis, there was a strong positive correlation between reticulated platelet quantity and MTT at baseline (R = 0.766 and R^2^ = 0.586, *p* < 0.001). There was also a strong positive correlation between reticulated platelet quantity and CD34 quantity (R = 0.602, and R^2^ = 0.362 *p* < 0.001), CD133 quantity (R = 0.666 and R^2^ = 0.4430, *p* < 0.001) and CFU levels (R = 0.437 and R^2^ = 0.191, *p* < 0.001).

After accounting for cofounders, the multiple linear regression showed that the MTT levels at baseline continue to have an independent correlation with RP (adjusted R^2^ = 0.793; *p* < 0.001). Diabetes mellitus was the only other factor significantly associated with MTT levels (adjusted R^2^ = 0.195; *p* = 0.017). CD 34 and CD133 levels also had a strong positive correlation with RP levels (adjusted R^2^ = 0.654; *p* < 0.001 and adjusted R^2^ = 0.627; *p* < 0.001, respectively). However, the levels of CD133 and CFU had a weak independent correlation with RP (adjusted R^2^ = 0.337; *p* = 0.008 and adjusted R^2^ = 0.195, *p* = 0.030, respectively).

At 24 h after PPCI, the correlation between RP and EPC markers was not significant, except for MTT (R = 0.465, R^2^ = 0.216, *p* < 0.001), as shown in Table 5.

A separate analysis was performed on the 38 (42.7%) patients who were taking aspirin at baseline, showing similar results: the levels of MTT (adjusted R^2^ = 0.701; *p* < 0.001), CD34^+^ and CD133^+^ (adjusted R^2^ = 0.638; *p* < 0.001 and adjusted R^2^ = 0.588; *p* < 0.001, respectively) had strong independent correlations with RP response. After 24 h, the correlation between RP quantity and EPC markers was significant only for MTT levels (R = 0.493, R^2^ = 0.341, *p* < 0.001).

## 4. Discussion

The main finding of our study is that, in patients with STEMI, higher levels of RP at baseline are significantly correlated with a more potent EPC response. This correlation was significant for several measured parameters of EPC function and quantity. After correcting for possible confounders, measured EPC markers, except for CFU levels, were still strongly associated with RP. A total of 24 h after admission and following PPCI, a weaker correlation still remained with MTT, but not with the other measurements of EPC.

ST-elevation myocardial infarction is the most acute and life-threatening presentation of coronary artery disease. The rupture of the atherosclerotic plaque and the formation of a platelet aggregate are the initiating events causing acute macrovascular occlusion of the coronary artery. This cascade of events involves a potent coagulation milieu, including increased platelet activation, the release of vasoactive substances and increased platelet aggregation. The treatment of this arterial milieu, and particularly platelets, is a key therapeutic target in the management of patients with STEMI.

There has been increasing research into platelet subpopulations —specifically, those of reticulated platelets. RP are younger, hyperactive platelets released in situations of increased platelet turnover. They have been shown to be increased in ischemic states such as those of acute coronary syndrome and stroke [10,11]. These platelets have increased volumes and a larger number of dense granules including mRNA. They have increased reactivity, including increased and persistent aggregation formation, compared to mature platelets [1,12]. This increased thrombotic potential has been shown to be of clinical importance, as RP have been associated with adverse cardiac events in multiple settings [13,14]. RP have been suggested as a prognostic marker of adverse cardiovascular events among patients with coronary artery disease [1]. Indeed, RP are strongly related to residual platelet reactivity despite prasugrel antiplatelet therapy. Our group previously investigated the association between RP and the response to prasugrel therapy amongst STEMI patients and found that those with higher RP levels had higher residual platelet reactivity despite antiplatelet therapy [15]. The ADAPT-DES trial investigated the relationship between platelet reactivity in patients treated with drug eluting stents and dual antiplatelet therapy (aspirin and clopidogrel) and clinical outcomes. They found that the high platelet reactivity on clopidogrel therapy was significantly related to the increased hazard ratios for stent thrombosis and myocardial infarction [16]. This relationship could potentially be explained by the residual platelet reactivity caused by the lower sensitivity of RP to certain antiplatelet therapies [17].

On the other hand, EPC have an important role in maintaining endothelial function and promoting vascular regeneration. EPC have also been shown to have prognostic value, and a reduced number of EPC has been associated with adverse events in a variety of atherosclerotic disease states such as coronary artery disease and peripheral vascular disease [18].

The biologic interaction between platelets and EPC is multifactorial and likely to represent a synergistic relationship. Platelet exposure itself has been shown to positively augment the EPC functional properties of migration, proliferation, differentiation and the production of nitric oxide metabolites in vitro [3]. In vitro studies have shown that the interaction between platelets and EPC occurs both in static and flow conditions [19].

Our study is the first to demonstrate a positive correlation between the young hyper-reactive population of RP and EPC response in vivo in the setting of STEMI, in which intense myocardial ischemia is present. Our findings demonstrated that a higher level of RP, representing a more intense thrombotic response, is also associated with a heightened EPC reaction that potentially acts to regenerate and restore endothelial integrity. Our findings add insight into the initial coronary vascular interactions during STEMI—that the acute event activates increased RP and EPC responses that strongly correlate and “crosstalk” biologically with each other.

The mechanism of this interaction is multifactorial, and multiple chemokines and growth factors have been implicated in this interplay [3,19].

Marfella et al., investigated the effect of glycemic control on EPC functioning in the setting of STEMI [7]. Previous data had shown that stress hyperglycemia was associated with impaired myocardial salvage in patients presenting with acute myocardial infarction [20]. Marfella et al., showed that glycemic control affected the EPC number and their ability to differentiate in the setting of STEMI [7]. Indeed, our findings showed that diabetes mellitus had an independent significant correlation with MTT, a marker of EPC functioning, at baseline STEMI presentation. Epigenetic factors have also been implicated in the STEMI coagulation cascade. D’Onofrio et al., showed that impaired sirtuin-1 expression was associated with increased thrombus burden in the setting of hyperglycemia STEMI patients. Reduced endothelial sirtuin-1 activity was associated with increased microRNA-33 and higher levels of inflammatory markers and oxygen reactive species [8].

Alexandru demonstrated the interplay in which EPC administration in animal models augmented EPC-platelet functioning in dynamic flow conditions [21]. Our findings add to this interplay by correlating RP with EPC function in vivo. The interaction between EPC and RP demonstrated in our study has important potential, albeit hypothesis-generating, implications. Platelet inhibition is an immediate therapeutic target in the setting of STEMI, and decreasing thrombus burden and microvascular obstruction have been associated with improved outcomes [22]. However, the subpopulation of RP play an important role in vascular regeneration. We found that the heightened EPC response, both in quantity and in function, remained, notwithstanding antiplatelet drug loading and PPCI. This is an important finding in that the use of antiplatelets and the ensuing attenuation of the thrombotic cascade do not impede on the EPC response directly.

Following PPCI and antiplatelet therapy, the EPC response remained augmented, but the correlation between the RP and EPC response was attenuated and only seen in MTT, a marker of EPC functioning. This suggests that the initial interaction between these cells not only has an immediate effect but may initiate a cascade of effects that act to restore vascular integrity.

Our study has some important limitations: it is observational in nature, and the correlation between RP and EPC quantity and function is not causal. Although we described a relationship between RP and EPC, this finding does not clarify the pathophysiological mechanism of this relationship, and further studies are warranted to reveal more concrete RP/EPC interactions. Our study was focused on the cellular interaction between RP and EPC, and we did not investigate the possible clinical sequelae of this interaction, as per clinical or imaging outcomes. Another limitation is that inflammatory markers were not investigated in this study cohort, and these markers may influence the interaction between RP and EPC. Our study included a relatively small number of patients, and our findings need to be validated in larger cohorts. Due to the small size of the cohort, we were unable to perform subgroup analysis to explore the interactions/effects of other baseline variables.

Nevertheless, this is the first study to demonstrate the strong correlation between RP and EPC in vivo in the acute setting of STEMI.

## 5. Conclusions

In patients with STEMI, higher levels of RP at baseline are significantly correlated with a more potent EPC response. Our finding needs further research and extended validation.

## Figures and Tables

**Table 1:** Baseline characteristics of the cohort.

Variable	All Patients *n* = 89	Prasugrel *n* = 41	Ticagrelor *n* = 42	Clopidogrel *n* = 6	*p* Value
Age (years)	61.9 ± 11.0	61.5 ± 11.3	61.3 ± 10.9	63.4 ± 11.8	0.48
Female sex (%)	30 (33.7)	11 (27)	16 (38)	3 (50)	0.14
BMI (kg/m^2^)	28.2 ± 4.3	28.7 ± 4.4	28.1 ± 4.5	27.9 ± 4.7	0.26
Smoking (%)	36 (40.4)	19 (46)	14 (33)	3 (50.0)	0.22
Diabetes mellitus (%)	35 (39.3)	16 (39)	16 (38)	3 (50.0)	0.42
Hypertension (%)	48 (53.9)	20 (49)	24 (57)	4 (66.7)	0.12
PVD (%)	6 (6.7)	2 (5)	3 (7.1)	1 (16.7)	0.27
CAD (%)	27 (30.3)	12 (29.3)	13 (31)	2 (33.3)	0.81
COPD (%)	14 (15.7)	7 (17)	6 (14)	1 (16.7)	0.70
LVEF (%)	49.1 ± 7.9	48.8 ± 8.1	50.4 ± 6.8	48.8 ± 8.1	0.34
Aspirin (%)	38 (42.7)	15 (36.6)	18 (42.9)	3 (50.0)	0.22
Statin (%)	51 (57.3)	23 (56.1)	24 (57.1)	4 (67.7)	0.62
Beta blocker (%)	21 (23.6)	10 (24.4)	9 (21.4)	2 (33.3)	0.75
ACEI (%)	31 (34.8)	14 (34.1)	15 (35.7)	2 (33.3)	0.88

Abbreviations: ACEI, angiotensin-converting enzyme inhibitor; CAD, coronary artery disease; COPD, chronic obstructive pulmonry disease; Hs, high sensetivity; LVEF, left ventricular ejection fraction; MPV, mean platelet volume; PVD, peripheral vascular disease; WBC, white blood cell. Variables presented as the mean ± standard deviation for continuous variables and as *n* (%) for categorical variables.

**Table 2:** Laboratory Results.

Test	Levels
Hemoglobin (g/dl)	14.3 ± 1.8
Platelet count (K/micl)	244.708
Mean platelet volume (fl)	9.672
White blood cell count (K/micl)	10.427
Creatinine (mg/dL)	0.912
Glucose (mg/dL)	165.461
Troponin T (median, (IQR)) (ng/L)	1205.5 (622.4–2857.2)
PRU Baseline	248.2 ± 48.8
PRU T1	18.9 ± 12.4
CFU Baseline (%)	1.22 ± 0.81
CFU T1 (%)	1.62 ± 0.90
CD34^+^ Baseline (%)	1.09 ± 0.83
CD34^+^ T1 (%)	1.96 ± 1.24
CD133^+^ Baseline (%)	0.88 ± 0.45
CD133^+^ T1 (%)	1.83 ± 1.17
MTT Baseline (%)	0.13 ± 0.08
MTT T1 (%)	0.54 ± 0.27
RP Baseline (%)	0.66 ± 0.23
RP T1 (%)	0.54 ± 0.28

PRU = Platelet Reactivity Unit; T1 = 24 h after primary percutaneous intervention; CFU = colony-forming units; MTT = 3-(4,5-dimethylthiazol-2-yl)-2,5-diphenyl tetrazolium bromide; RP = reticulated platelet. Variables presented as the mean ± standard deviation for continuous variables and as *n* (%) for categorical variables.

**Table 3:** Rates of EPC by RP Tertiles.

Measurement	RP Tertile 1	RP Tertile 2	RP Tertile 3	*p* Value
CFU	0.52	1.29	1.63	<0.01
CD133^+^ (%)	0.30	0.63	1.65	0.02
CD34^+^ (%)	0.42	0.96	1.88	<0.01
MTT (%)	0.08	0.11	0.20	0.04

CFU = colony-forming units; MTT = 3-(4,5-dimethylthiazol-2-yl)-2,5-diphenyl tetrazolium bromide; RP = reticulated platelet.

**Table 4:** Correlation between RP and EPC markers at baseline assessment.

		RP	MTT	CFU	CD34^+^	CD133^+^
RP	Pearson Correlation	1	0.766	0.437	0.602	0.666
	*p* value		<0.001	<0.001	<0.001	<0.001
	*n*	89	89	85	85	84
MTT	Pearson Correlation	0.766	1	0.401	0.537	0.519
	*p* value	<0.001		<0.001	<0.001	<0.001
	*n*	89	89	85	85	84
CFU	Pearson Correlation	0.437	0.401	1	0.324	0.461
	*p* value	<0.001	<0.001		0.003	<0.001
	*n*	85	85	85	81	81
CD34^+^	Pearson Correlation	0.602	0.537	0.324	1	0.694
	*p* value	<0.001	<0.001	0.003		<0.001
	*n*	85	85	81	85	83
CD133^+^	Pearson Correlation	0.666	0.519	0.461	0.694	1
	*p* value	<0.001	<0.001	<0.001	<0.001	
	*n*	84	84	81	83	84

CFU = colony-forming units; MTT = 3-(4,5-dimethylthiazol-2-yl)-2,5-diphenyl tetrazolium bromide; RP = reticulated platelet.

**Table 5:** Pearson correlation between RP and EPC markers 24 h after PPCI.

		RP
CD34^+^	Pearson Correlation	0.260
	*p* value	0.073
CD133^+^	Pearson Correlation	0.133
	*p* value	0.796
MTT	Pearson Correlation	0.465
	*p* value	<0.001
CFU	Pearson Correlation	0.206
	*p* value	0.159

CFU = colony-forming units; MTT = 3-(4,5-dimethylthiazol-2-yl)-2,5-diphenyl tetrazolium bromide; RP = reticulated platelet.

## Data Availability

The data presented in this study were done at Rabin Medical Center and are not publicly available.
